# Risk of recurrent spontaneous preterm birth following preterm full dilatation cesarean delivery

**DOI:** 10.1111/aogs.70115

**Published:** 2026-01-19

**Authors:** Amrita Banerjee, Agnieszka Glazewska‐Hallin, Maria Ivan, Tatiana Nazarenko, Charlotte Colley, Natalie Suff, Lisa Story, Davide Casagrandi, Amos Tetteh, Natalie Greenwold, Manju Chandiramani, Jenny Carter, Raffaele Napolitano, Andrew H. Shennan, Anna L. David

**Affiliations:** ^1^ Fetal Medicine Unit, Elizabeth Garrett Anderson Wing University College London Hospital London UK; ^2^ Elizabeth Garrett Anderson Institute for Women's Health University College London London UK; ^3^ Department of Women's and Children's Health St Thomas' Hospital, King's College London London UK; ^4^ Department of Mathematics University College London London UK; ^5^ National Institute for Health and Care Research University College London Hospitals Biomedical Research Centre London UK

**Keywords:** full dilatation cesarean delivery, preterm birth, transvaginal ultrasound

## Abstract

**Introduction:**

Term full dilatation cesarean delivery (FDCD) is associated with an increased risk of subsequent spontaneous preterm birth (sPTB). The impact of preterm FDCD on recurrent sPTB is unknown. We investigated the relationship between recurrent sPTB and the mode of prior sPTB.

**Material and Methods:**

This is a retrospective cohort study of singleton pregnant women attending two high‐risk preterm birth surveillance clinics (University College London Hospital and St Thomas' Hospital London, UK), with one previous sPTB (24–36 + 6 weeks). Women were categorized according to their mode of birth in the index sPTB pregnancy: (1) preterm FDCD, (2) preterm vaginal birth and (3) preterm cesarean delivery at <10 cm cervical dilatation (CD < 10 cm). The primary outcome was recurrent sPTB <37 weeks of gestation. Secondary outcomes included sPTB <34 weeks, <28 weeks, spontaneous late miscarriage and short cervical length (≤25 mm). In a subgroup of women with preterm FDCD, CD scar characteristics were assessed during the second trimester of pregnancy using transvaginal ultrasound.

**Results:**

Median gestation of prior sPTB was similar across all groups (32 weeks; *p* = 0.454). Recurrent sPTB <37 weeks was significantly more common in women with previous preterm FDCD, 38.1% (8/21) compared to vaginal birth, 15.1% (16/106) or CD < 10 cm, 13.8% (15/109); aOR 4.4 (95% CI 1.3–14.9; *p* = 0.023) and 5.1 (95% CI 1.6–16.5; *p* = 0.022), respectively. Recurrent sPTB <34 weeks was even higher in the previous preterm FDCD group, 23.8% (5/21) compared to vaginal birth 4.7% (5/106) or CD < 10 cm 8.3% (9/109); aOR 16.6 (95% CI 2.8–97.2; *p* = 0.016) and 5.7 (95% CI 1.4–23.1; *p* = 0.022), respectively. CD scar location was assessed in 15 women with preterm FDCD in one centre. Scar visualization was 87%, with 77% (10/13) of scars being located within the cervix or <5 mm above the internal cervical os.

**Conclusions:**

Women undergoing FDCD following preterm labor have a significantly higher risk of recurrent sPTB at <37 and <34 weeks of gestation compared to women with previous preterm vaginal birth or CD prior to the second stage of labor. These findings suggest that preterm FDCD may further compromise cervical function. It is important that clinicians are aware of this increased risk of recurrent sPTB to guide patient counseling and management accordingly.

## Graphical abstract

Women with a history of spontaneous preterm labor resulting in full dilatation cesarean delivery have a higher risk of recurrent spontaneous preterm birth than those with prior preterm vaginal birth or first stage cesarean delivery. Clinicians should be aware of these risks to guide counseling and management in subsequent pregnancies.

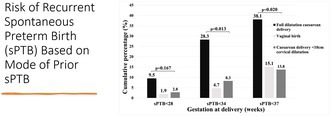

**Abbreviations**
CD: cesarean delivery
CD < 10 cm: cesarean delivery at <10 cm cervical dilatation
FDCD: full dilatation cesarean delivery
PTB: preterm birth
sPTB: spontaneous preterm birth

**Key message**

Prior full dilatation cesarean delivery following spontaneous preterm labor carries a higher risk of recurrent spontaneous preterm birth compared to prior preterm vaginal birth or cesarean during the first stage of labor.

## 1 INTRODUCTION

Preterm birth (PTB), delivery before 37 weeks of gestation, is a major global health burden.^1^ Globally, there has been little change in the PTB rate over the last decade, estimated at 9.8% and 9.9% of all livebirths in 2010 and 2020, respectively.^2^ In multiparous women, previous spontaneous PTB (sPTB) is the single most important risk factor for the prediction of subsequent sPTB, with the risk being inversely related to the gestation of previous sPTB.^3^ There is uncertainty about the potential impact of the mode of preterm birth on maternal and neonatal outcomes and no information on the associated risk of recurrent sPTB.^4^, ^5^ NHS Maternity Statistics in England report that in 2022–2023, cesarean delivery (CD) was performed in 4.7% of births at ≤31 weeks gestation and in 8.8% of births between 32 and 36 weeks gestation.^6^

Emerging evidence from various studies has shown a significant association between previous term (≥37 weeks gestation) CD and subsequent sPTB, especially when undertaken at full cervical dilatation (10 cm cervical dilation).^7^, ^8^, ^9^, ^10^, ^11^, ^12^, ^13^, ^14^ Worryingly, this cohort of women is also at an increased risk of recurrent sPTB.^14^, ^15^, ^16^ The mechanism of sPTB following a previous term full dilatation CD (FDCD) is likely due to cervical trauma.^17^ In a recent prospective cohort study of pregnant women with previous term FDCD, a low CD scar position (within the cervix or <5.0 mm above the internal cervical os) was associated with an increased risk of shortening cervical length and/or sPTB, aOR 12.7, 95% CI 4.5–36.0; *p* ≤ 0.0001.^17^ Whilst national maternity care programs, such as the NHS England Saving Babies Lives Care Bundle 3, recommend preterm birth surveillance for women with previous term FDCD, the magnitude of the risk of recurrent sPTB associated with a preterm FDCD is yet to be elucidated.^18^ We hypothesized that due to the additional intraoperative complexities, there would be a cumulative impact of FDCD in combination with a sPTB and the rate of recurrent sPTB in these women would be significantly higher than in women with either a spontaneous preterm vaginal birth or CD at <10 cm cervical dilatation. This is important information for patient counseling and may help select interventions to prevent recurrent sPTB.

## 2 MATERIAL AND METHODS

This is a retrospective cohort study of singleton pregnant women attending two high‐risk preterm birth surveillance clinics (University College London Hospital and St Thomas' Hospital London, UK). All participants were identified through the UK Preterm Clinical Network (UKPCN) Database (REC Ref. 22/ES/0001).^19^ The database search included women who had attended the preterm birth surveillance clinics between October 2010 and March 2024.

All women included in this study had a history of one previous sPTB between 24 and 36 + 6 weeks gestation and a subsequent pregnancy that reached at least 14 weeks' gestation. Women with a previous history of spontaneous late miscarriage (spontaneous loss of intrauterine pregnancy between 14 and 23 + 6 weeks) or spontaneous livebirth prior to 24 weeks' gestation in the initial pregnancy were excluded as no CD was undertaken prior to 24 weeks' gestation. Women were excluded if they had a termination of pregnancy (e.g. for fetal anomaly), iatrogenic preterm birth and pre‐pregnancy transabdominal cerclage. Women who had sPTB prophylactic interventions (vaginal cervical cerclage and/or vaginal progesterone) during the subsequent pregnancy were included in the study. If a woman had more than one pregnancy during the study period, only the first pregnancy was included from the database.

Women were divided into three cohorts according to their previous mode of sPTB: preterm FDCD, preterm vaginal birth and preterm CD at <10 cm cervical dilatation (CD < 10 cm). The vaginal birth cohort did not include any women with a history of previous CD and both the CD groups only included women with one previous CD (undertaken during the previous preterm birth). Data obtained from the UKPCN database included demographic characteristics, risk factors for sPTB, previous obstetric history, clinic visits and surveillance test results, PTB interventions and outcomes.

The primary outcome was sPTB <37 weeks gestation. Secondary outcomes included sPTB further categorized by gestational week of birth: <34 weeks, <28 weeks and spontaneous late miscarriage (spontaneous loss of intrauterine pregnancy between 14 and 23 + 6 weeks). Other outcomes analyzed included mid‐trimester (14–24 weeks gestation) shortening of cervical length (≤25 mm) and sPTB prophylactic interventions e.g. vaginal progesterone and/or vaginal cervical cerclage. At both hospitals women underwent serial transvaginal ultrasound assessment of cervical length between 14 and 24 weeks gestation at two to three weekly intervals. Prophylactic interventions (vaginal cerclage and/or vaginal progesterone) were offered for short cervical length (≤25 mm) or as per UK RCOG and NICE guidelines.^20^, ^21^ The shortest cervical length during mid‐trimester screening was used for analysis.

A subgroup of the overall preterm FDCD cohort identified from the UKPCN database who were booked at University College London's PTB surveillance clinic also underwent prospective assessment of CD scar location in relation to the internal cervical os and scar niche characteristics as part of routine clinical surveillance between 14 and 24 weeks gestation. This was performed using a validated transvaginal ultrasound technique, without saline or gel contrast enhancement (Voluson E8 or E10 Expert ultrasound system, GE Healthcare, Zipf, Austria, 4–9‐MHz transvaginal probe).^22^ Senior clinicians who were trained in standardized transvaginal ultrasound identification and measurements of CD scar characteristics performed the assessments. The CD scar was defined as a hypoechogenic (or rarely hyperechogenic) discontinuity of the myometrium at the anterior wall of the lower uterine segment or cervix. A niche was defined as an indentation at the CD scar site with a depth of ≥2 mm. The shortest distance from the CD scar base to the internal cervical os was recorded in the sagittal plane.

The relative position of the CD scar from the internal cervical os was defined as the anatomical distance in millimeters above (+mm) or millimeters below (−mm) the internal os. Niche parameters (length, depth, width, residual and adjacent myometrial thickness) were recorded in sagittal and transverse planes, accordingly. In the presence of a niche, CD scar position was recorded as the shortest distance from the edge of the niche base to the internal cervical os. The most distal recorded position of the CD scar to the internal cervical os was used for analysis.

Statistical analysis was performed using SPSS version 29.0 (SPSS Inc., Chicago, IL, USA).

Data from categorical variables were presented as numbers and percentages and continuous variables were expressed as median and interquartile range (IQR). Comparison of categorical variables were performed using chi‐squared and Fisher exact tests. Normality of continuous variables was checked using the Shapiro–Wilk test. All continuous variables did not have a normal distribution. Comparison of continuous variables were analyzed using Mann–Whitney *U* or Kruskal–Wallis test accordingly. The Kaplan–Meier plot with Log‐rank (Mantel–Cox) test was used for comparison of gestational age at delivery. Multivariable logistic regression analysis was undertaken to adjust for clinically relevant sPTB risk factors (ethnicity, previous sPTB gestation, history of preterm prelabor rupture of membranes (PPROM), uterine anomaly and cervical surgery). For missing values of demographic data, one BMI measurement was missing in the whole cohort, the missing value was replaced with the overall mean. Adjusted odds ratio (aOR) with 95% confidence interval (95% CI) were calculated for the sPTB outcome measures. A *p*‐value of less than 0.05 was considered statistically significant. For subgroup analysis, *p*‐values were adjusted for multiple comparisons using the Benjamini‐Hochberg procedure.

Currently the risk of recurrent sPTB following a preterm FDCD is not known. Therefore, from existing published evidence, we estimated that the risk of subsequent sPTB following a preterm vaginal birth would be 20% and following a preterm FDCD to be 50%.^14^ We considered that FDCD in preterm labor is less common than CD < 10 cm or vaginal birth. Therefore, we calculated that 21 participants in the preterm FDCD and 105 participants in the preterm vaginal birth group would be required to detect significant differences with a power of 80%. We aimed to include a similar number of preterm CD < 10 cm women as the preterm vaginal birth cohort as we estimated their risk of subsequent sPTB would be lower compared to the preterm FDCD cohort. The final numbers included in the preterm FDCD and preterm CD < 10 cm cohorts were from the whole UKPCN database search which corresponded to our estimated participant numbers. To ensure the preterm vaginal birth cohort included recent contemporaneous patients, we limited the inclusion criteria to a 3‐year period (2017–2019) as a comparison reference group which was in keeping with our power calculation estimates.^19^

## 3 RESULTS

**FIGURE 1:**
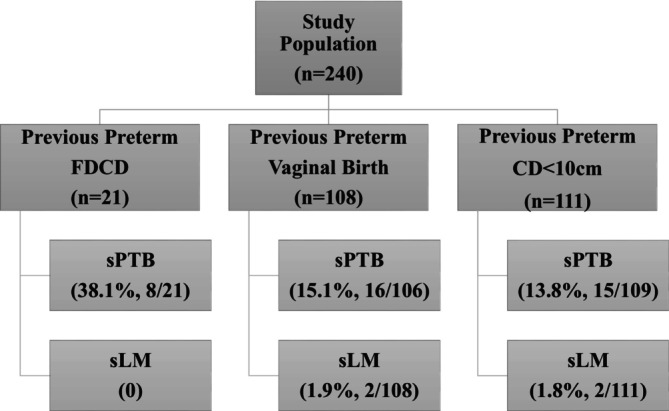
Flowchart of study population. CD < 10 cm, cesarean delivery at <10 cm cervical dilatation; FDCD, full dilatation cesarean delivery; sLM, spontaneous late miscarriage; sPTB, spontaneous preterm birth.

We identified 240 women from the database with one previous sPTB and complete primary outcome data from a subsequent pregnancy: 21 women with previous preterm FDCD, 108 women with previous preterm vaginal birth and 111 women with previous preterm CD < 10 cm. The study flowchart is shown in Figure 1.

Maternal demographic and previous obstetric characteristics of the study population are summarized in Table 1. Demographic characteristics were generally similar between the groups and the median gestation of the previous sPTB was similar across all three groups: preterm FDCD 32 (range 25–36), preterm vaginal birth 32 (range 24–36) and preterm CD < 10 cm 32 (range 24–36) weeks' gestation, *p* = 0.454. Previous extreme sPTB prior to 27 weeks' gestation occurred in: 9.5% (2/21), preterm FDCD; 18.5% (20/108), preterm vaginal birth; and 11.7% (13/111), preterm CD < 10 cm, *p* = 0.285, respectively.

**TABLE 1:** Demographic characteristics of the study population compared with previous modes of spontaneous preterm birth.

Background characteristics	Mode of previous spontaneous preterm birth			*p*‐value
	FDCD (*n* = 21)	Vaginal birth (*n* = 108)	CD < 10 cm (*n* = 111)	
Maternal age, years	34.0 (31.5–35.5)	34.0 (30.0–37.0)	35.0 (31.0–38.0)	0.313
BMI, kg/m^2^	22.9 (20.6–27.0)	23.4 (21.5–28.4)	25.1 (21.9–28.6; *n* = 110)	0.245
Ethnicity				
White	42.9 (9)	58.3 (63)	54.1 (60)	0.411
Black	9.5 (2)	24.1 (26)	26.1 (29)	0.259
Other	47.6 (10)	17.6 (19)	19.8 (22)	0.008
Current smoker	0 (0)	8.3 (9)	2.7 (3)	0.088
Parity				
1	76.2 (16)	73.1 (79)	72.1 (80)	0.925
≥2	23.8 (5)	26.9 (29)	27.9 (31)	
Previous sPTB gestation (weeks)	32 (31–35)	32 (27–35)	32 (29–35)	0.454
Previous PPROM	42.9 (9)	23.1 (25)	52.3 (58)	<0.001
Cervical surgery	9.5 (2)	13.9 (15)	10.8 (12)	0.730
Uterine anomaly	4.8 (1)	5.6 (6)	9.0 (10)	0.554

*Note:* Data are given as % (n) or median (IQR‐interquartile range).

Abbreviations: BMI, body mass index; CD < 10 cm, cesarean delivery at <10 cm cervical dilatation; FDCD, full dilatation cesarean delivery; PPROM, preterm prelabor rupture of the membranes; sPTB, spontaneous preterm birth.

**FIGURE 2:**
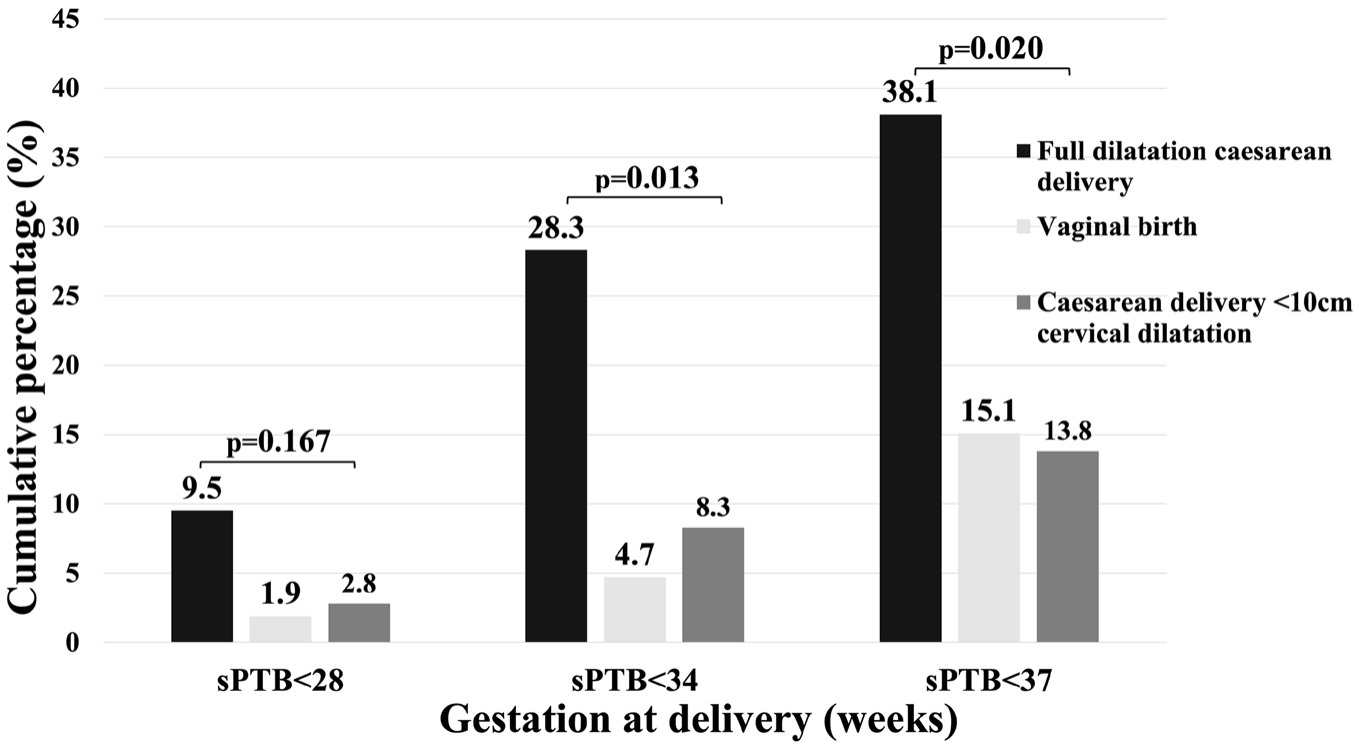
Recurrent spontaneous preterm birth according to mode of previous. sPTB, spontaneous preterm birth.

Overall, recurrent sPTB occurred in significantly more women with a previous preterm FDCD, 38.1% (8/21) compared to women with a preterm vaginal birth, 15.1% (16/106) or preterm CD < 10 cm, 13.8% (15/109), OR 3.5 (95% CI 1.2–9.7; *p* = 0.032) and OR 3.9 (95% CI 1.4–10.9; *p* = 0.022), respectively (Tables 2 and 3). Following adjusting for potential confounders the risk of sPTB at <37 weeks' gestation following a preterm FDCD was even higher (Table 3): preterm FDCD compared to preterm vaginal birth, aOR 4.4 (95% CI 1.3–14.9; *p* = 0.023) and preterm FDCD compared to preterm CD < 10 cm, aOR 5.1 (95% CI 1.6–16.5; *p* = 0.022). The risk of subsequent sPTB was noted to be the highest at <34 weeks' gestation in women with a previous preterm FDCD compared to a previous preterm vaginal birth, 23.8% (5/21) versus 4.7% (5/106), OR 6.3 (95% CI 1.6–24.3; p = 0.022) and aOR 16.6 (95% CI 2.8–97.2; *p* = 0.016), Figure 2. All sPTBs occurred between 23 and 36 weeks gestation. Spontaneous late mid‐trimester miscarriage occurred in four women (between 17 and 22 weeks gestation): two women with a previous preterm vaginal birth, (1.9%, 2/108) and two women with a previous preterm CD < 10 cm, (1.8%, 2/111).

**TABLE 2:** Rates of recurrent spontaneous preterm birth and shortening cervical length compared with previous mode of spontaneous preterm birth.

	Mode of previous spontaneous preterm birth			*p*‐value
	FDCD (*N* = 21)	Vaginal birth (*N* = 108)	CD < 10 cm (*N* = 111)	
Primary outcome				
sPTB < 37 weeks	38.1 (8/21)	15.1 (16/106)	13.8 (15/109)	0.020
Secondary outcomes				
sPTB < 34 weeks	23.8 (5/21)	4.7 (5/106)	8.3 (9/109)	0.013
sPTB < 28 weeks	9.5 (2/21)	1.9 (2/106)	2.8 (3/109)	0.167
sLM	0 (0)	1.9 (2/108)	1.8 (2/111)	0.822
sLM or sPTB < 37 weeks	38.1 (8)	16.7 (18)	15.3 (17)	0.040
Overall gestation of subsequent births, weeks	38.0 (34.7–39.3)	38.6 (37.4–39.7)	38.9 (37.4–39.9)	0.115
CL ≤ 25 mm	23.8 (5/21)	18.8 (19/101)	12.2 (12/98)	0.285
Gestational age at CL ≤ 25 mm, weeks	17.6 (15.3–22.0, *n* = 5)	20.3 (16.4–23.6, *n* = 17)	19.9 (18.5–23.5, *n* = 12)	0.476

*Note*: Data are given as % (*n*). or %(*n*/*N*) or median (IQR‐interquartile range).

Abbreviations: CD < 10 cm, cesarean delivery at <10 cm cervical dilatation; CI, confidence interval; CL, cervical length; FDCD, full dilatation cesarean delivery; sLM, spontaneous late miscarriage (14–22 weeks gestation); sPTB, spontaneous preterm birth (≥23 weeks gestation).

**TABLE 3:** Odds ratios of recurrent spontaneous preterm birth by mode of prior spontaneous preterm birth.

Subsequent pregnancy outcome	Mode of previous spontaneous preterm birth											
	FDCD vs. vaginal birth						FDCD vs. CD < 10 cm					
	OR (95% CI)	*p*‐value	*p*‐value (adj)^a^	aOR (95% CI)^b^	*p*‐value	*p*‐value (adj)^b^	OR (95%CI)	*p*‐value	*p*‐value (adj)^a^	aOR (95% CI)^b^	*p*‐value	*p*‐value (adj)^a^
sPTB												
<37 weeks	3.5 (1.2–9.7)	0.028	0.032	4.4 (1.3–14.9)	0.017	0.023	3.9 (1.4–10.9)	0.013	0.022	5.1 (1.6–16.5)	0.006	0.022
<34 weeks	6.3 (1.6–24.3)	0.011	0.022	16.6 (2.8–97.2)	0.002	0.016	3.5 (1.0–11.7)	0.051	0.051	5.7 (1.4–23.1)	0.014	0.022

*Note*: There was no significant difference between the preterm CD < 10 cm and preterm vaginal birth groups.

Abbreviations: aOR adjusted odds ratio; CD < 10 cm, cesarean delivery at <10 cm cervical dilatation; CI, confidence interval. FDCD, full dilatation cesarean delivery; sPTB, spontaneous preterm birth.

^a^ False discovery rate (FDR) adjusted *p* value (Benjamini–Hochberg procedure).

^b^ Adjusted for ethnicity, gestational age of previous sPTB, previous PPROM (preterm prelabor rupture of membranes), history of cervical surgery and uterine anomaly.

**FIGURE 3:**
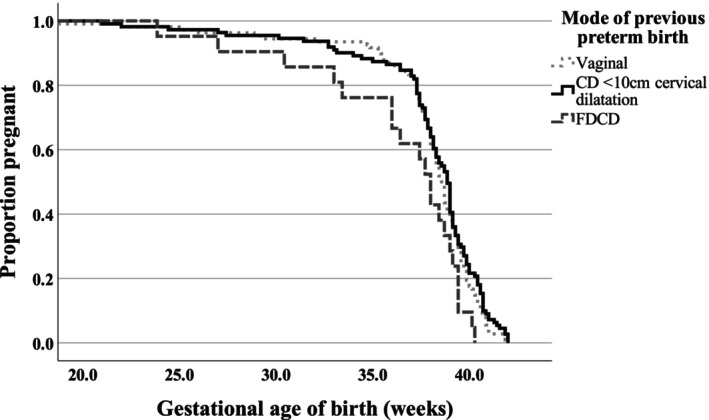
Kaplan Meier survival curve demonstrating the outcome of the subsequent pregnancy according to the mode of previous spontaneous preterm birth. CD, cesarean delivery; FDCD, full dilatation cesarean delivery. On log rank (Mantel–Cox) test *p* = 0.033.

The overall median gestation of subsequent birth in women with previous preterm FDCD, preterm vaginal birth and preterm CD < 10 cm was 38.0 (interquartile range, IQR 34.7–39.3), 38.6 (IQR 37.4–39.7) and 38.9 (IQR 37.4–39.9) weeks, *p* = 0.115. Gestational age of birth in relationship with previous mode of sPTB is further demonstrated in the Kaplan Meier survival plot (*p* < 0.033), Figure 3. Women who had recurrent sPTB, the median gestation of subsequent sPTB was noted to be 2.1 weeks later compared to the index sPTB following a previous preterm vaginal birth (35.1 weeks versus 33.0 weeks), while in women with previous preterm FDCD the gestation of recurrent sPTB was similar to the index sPTB (33.2 weeks versus 33.0 weeks), Table 4. However, these differences in gestational age of birth did not reach statistical significance. Mid‐trimester shortening of cervical length (≤25 mm) occurred in 23.8% (5/21), 18.8% (19/101) and 12.2% (12/98) of women with previous preterm FDCD, preterm vaginal birth and preterm CD < 10 cm and this occurred at a median gestation of 17.6 (IQR, 15.3–22.0), 20.3 (IQR, 16.4–23.6) and 19.9 (IQR, 18.5–23.5) weeks, respectively.

**TABLE 4:** Comparison of gestation age of birth in women with recurrent spontaneous preterm birth.

Mode of previous sPTB	*N*	Women with recurrent sPTB		
		Gestational age of previous sPTB (weeks)	Gestational age of recurrent sPTB^a^ (weeks)	*p* value^b^
FDCD	8	33.0 (30.3–35.5)	33.2 (27.9–36.0)	0.611
Vaginal Birth	18	33.0 (28.5–35.0)	35.1 (28.1–35.8)	0.449
CD < 10 cm	17	32.0 (29.5–34.0)	32.7 (27.2–34.7)	0.641

*Note*: Data are given as % (*n*/*N*) or median (IQR‐interquartile range). Abbreviations: CD < 10 cm, cesarean delivery at <10 cm cervical dilatation; FDCD, full dilatation cesarean delivery; sPTB, spontaneous preterm birth.

^a^ Includes spontaneous late miscarriage.

^b^ Wilcoxon signed rank test.

Preterm birth prophylactic intervention (vaginal progesterone and/or vaginal cervical cerclage) rates were 38.1% (8/21), 25.9% (28/108) and 16.2% (18/111) in women with previous preterm FDCD, preterm vaginal birth and preterm CD < 10 cm, respectively, Table S1. In women receiving a prophylactic preterm birth intervention, the rate of spontaneous late miscarriage or sPTB was not significantly different between the groups; however there was a tendency towards a higher rate in the preterm FDCD cohort, Table S1. 62.5% (5/8) of women with a prior preterm FDCD had a sPTB despite a prophylactic preterm birth intervention (vaginal cerclage and/or vaginal progesterone). The analysis was limited due to the overall small numbers.

In the second part of our study, we analyzed the CD scar and niche characteristics in a subgroup of women with one previous transverse lower segment preterm FDCD (*n* = 15). Background demographics and CD scar characteristics are shown in Table S2. The CD scar was assessed at a median gestation of 17.1 (IQR, 15.8–18.4) weeks (Table S3). CD scar visualization was 86.7% (13/15) and a CD scar niche was present in 23.1% (3/13). The preterm FDCD scar was located within the cervix or <5 mm above the internal cervical os in 76.9% (10/13). The <5 mm distance cut off was used as this was identified as an optimal cut off in a prospective cohort study for prediction of sPTB in women with previous term FDCD.^17^ In this small cohort of women with previous preterm FDCD, 7/10 (70%) of women who had a CD scar within the cervix or <5 mm above the level of the internal cervical os experienced mid‐trimester shortening of CL (≤25 mm) and/or sPTB compared to 0/3 (0%) women with a CD scar that was noted higher up the uterus, *p* = 0.070. In the two women where the preterm CD scar was not visualized both women had a term birth.

## 4 DISCUSSION

In this study we investigated the relationship between recurrent sPTB and mode of prior sPTB with particular focus on women having had a preterm FDCD. In pregnancies following a previous sPTB, the risk of recurrent sPTB was significantly higher in women who underwent a FDCD following spontaneous preterm labor compared to a vaginal birth or CD at <10 cm cervical dilatation. The risk of recurrent sPTB was even higher at <34 weeks gestation compared to <37 weeks gestation. Using a validated transvaginal ultrasound technique, in a small cohort of women with preterm FDCD, we found that 77% of CD scars were located within the cervix or very close to the internal cervical os (<5 mm above it). Furthermore, in this preterm FDCD cohort, 70% of the women with a scar located within the cervix or close to the internal os experienced a subsequent mid‐trimester shortening of CL (≤25 mm) and/or sPTB or spontaneous late miscarriage.

The risk of recurrent sPTB following a term CD, including term FDCD has been documented in several studies.^14^, ^15^, ^16^ This study provides further evidence that FDCD even when performed following spontaneous preterm labor leads to an increased risk of sPTB in future pregnancies. In our study, despite a prophylactic preterm birth intervention (vaginal cerclage and/or vaginal progesterone) 62.5% (5/8) of women with a prior preterm FDCD had an sPTB. Other studies have reported that transvaginal cerclage may not be as effective for preterm prophylaxis in women with previous FDCD.^15^, ^16^ Hickland et al. reported that in women with previous FDCD, 35% delivered at <30 weeks' gestation following a vaginal cerclage.^16^

A history of sPTB and a prior FDCD are both established independent risk factors for subsequent sPTB. The mechanisms underlying these risks may overlap and potentially interact. Spontaneous onset of preterm labor may reflect underlying biological and structural susceptibilities that predispose women to recurrent preterm delivery. In contrast, the increased risk following FDCD is assumed to be due to iatrogenic trauma from the CD incision being close to the internal cervical os. When these risk factors coexist, the cumulative risk of sPTB may be additive or potentially synergistic. There is increasing evidence to support the concept of an active sphincter at the internal cervical os.^23^, ^24^ Therefore, damage to this sphincter can compromise its function to maintain a long, closed cervix in subsequent pregnancies. This is supported by the findings from a prospective cohort study assessing CD scar characteristics in women with previous term FDCD, where a scar located within <5 mm of the internal os had an almost sevenfold increased risk of subsequent sPTB (aOR 6.87; 95% CI 1.34–58; *p* = 0.035).^17^ Our initial findings suggest that transvaginal ultrasound assessment of preterm CD scar characteristics may be helpful in identifying women most at risk and could select those who would benefit most from prophylactic interventions.

Overall CD rates are rising globally, along with an increase in FDCD.^25^, ^26^ This upward trend is likely multifactorial, influenced by evolving standards in professional training and clinical practice, heightened medicolegal awareness, and changing social and cultural expectations.^25^ There remains significant uncertainty regarding the optimal mode of preterm birth especially in malpresentation such as breech. Professional guidelines based on consensus or limited evidence vary in their recommendations. In the UK, current guidance advises against routine CD for singleton preterm breech presentation in labor before 26 weeks' gestation,^27^ but recommends CD for preterm breech presentation when delivery is indicated due to maternal or fetal compromise. In contrast, US guidelines suggest that CD may be considered from 23 weeks' gestation in cases of malpresentation.^28^ Preterm labor can progress rapidly to full dilatation and the preterm fetus is more likely to be compromised during labor. International guidelines recommend caution with instrumental birth; therefore CD in the late first or second stage of labor is not uncommon. In the absence of robust evidence, the mode of delivery should be determined by an experienced obstetrician following comprehensive clinical assessment and in consultation with the woman. Preventing sPTB following an FDCD in subsequent pregnancies necessitates the development of cesarean incision, delivery, and repair techniques that minimize iatrogenic cervical injury, alongside potential re‐evaluation of intrapartum management strategies to reduce the incidence of FDCD. The unpredictable nature of preterm labor, however, poses significant challenges. High‐quality data from prospective studies is urgently needed to evaluate the long‐term maternal and neonatal outcomes in relation to mode of birth.

A key strength of this multi‐center study is the use of data from two long‐established high‐risk tertiary level preterm birth surveillance clinics, which follow comparable antenatal screening and management protocols serving large inner‐city populations. All data was collected according to the UKPCN database protocol reducing the likelihood of missing additional confounding risk factors.^19^ To the best of our knowledge, we are not aware of any other studies specifically comparing the risk of recurrent sPTB following preterm FDCD. Given the retrospective nature of the study however, there are some limitations. There is likely to be a degree of selection bias that could affect the risk estimates. Clinicians became aware of the sPTB risks associated with FDCD in the last 10 years since 2015 following the publication by Levine et al.^10^ Details of preceding cesarean delivery may not have been sought for all women and so to minimize potential bias only women with a fully documented previous obstetric history including the mode of previous deliveries were included. Furthermore, the strict inclusion criteria to minimize bias limited the sample size of the CD groups and the study was not powered to detect certain differences identified between groups e.g., the effect of preterm birth prophylactic interventions. It is plausible this also had an impact on our overall sPTB rates.

Although our data has shown an association between the mode of previous sPTB and the risk of recurrent sPTB, this requires validation in larger multicenter prospective cohort studies. We acknowledge that recruitment from two specialized PTB surveillance centers may limit generalizability to general obstetric populations. However, both centers serve geographically diverse populations and maintain comprehensive, standardized data collection protocols enhancing data quality. The risk of sPTB following a term CD appears to be a continuum with progressive cervical dilatation.^7^, ^10^, ^11^ Unfortunately, cervical dilatation data were unavailable in the CD < 10 cm cohort. Further large prospective studies are required to assess the CD scar characteristics in relation to the gestation of CD, cervical dilatation at the time of CD and its association with subsequent pregnancy outcomes. Postnatal assessment of CD scars has also reported an increase in CD scar niche prevalence following peripartum infection, fever and premature rupture of membranes.^29^, ^30^, ^31^, ^32^, ^33^ It is hypothesized that infection may affect the wound healing process and infection and inflammation often play a key role in preterm rupture of membranes and preterm labor.^34^ Therefore, in future studies assessing the risk factors associated with niche development following preterm FDCD would also be important.

Assessing the CD scar characteristics using transvaginal ultrasound from the preconception period through to the subsequent pregnancy could help identify key characteristics that can be used to develop multiparameter screening models. Early identification of women most at risk of sPTB is needed to ensure women at high risk are stratified accordingly to appropriate antenatal surveillance and prophylactic management options. Furthermore, prophylactic preventative measures (cervical cerclage and vaginal progesterone) for sPTB employed in current clinical practice, have not been investigated in prospective studies specifically in this population of women.

## 5 CONCLUSION

In conclusion, women undergoing an FDCD in spontaneous preterm labor have a significantly higher risk of recurrent sPTB at <37 and < 34 weeks gestation compared to women with a previous vaginal sPTB or preterm CD prior to second stage of labor. These findings suggest that a preterm FDCD likely compromises cervical function. It is important that clinicians are aware of this increased risk of recurrent sPTB and arrange appropriate preconception counseling and antenatal surveillance with interventions to prevent recurrence.

## AUTHOR CONTRIBUTIONS

Conceptualization: JC, RN, AHS, ALD. Data curation: AB, AG‐H, JC. Formal analysis: AB, AG‐H, TN. Funding acquisition: ALD, AHS, JC. Investigation: AB, AG‐H, MI, CC, NS, LS, DC, AT, NG, MC, JC, RN, AHS, ALD. Methodology: AB, AG‐H, TN, JC, RN, AHS, ALD. Project administration: JC, RN, AHS, ALD. Software: JC, AHS. Resources: JC, AHS, RN, ALD. Supervision: RN, AHS, ALD. Validation: AB, AG‐H, JC. Visualization: AB. Writing—original draft: AB, AG‐H. Writing—review and editing: all authors.

## FUNDING INFORMATION

This study was supported by Tommy's Charity at the Tommy's National Centre for Preterm Research. ALD is supported by the National Institute for Health and Care Research University College London Hospitals Biomedical Research Centre, London, UK. AB and MI were part supported by a grant from the Jon Moulton Charitable Foundation.

## CONFLICT OF INTEREST STATEMENT

ALD reports receiving consulting fees and is a member of the advisory board for Prena™, a company that is developing a device to reduce contractions leading to preterm birth. All other authors declare no competing interests.

## ETHICS STATEMENT

This project was undertaken under the ethical approval given on September 15, 2016 for the PCN Database, under REC Ref. 22/ES/0001 by the East of Scotland Research Ethics Service REC. The project application was approved by the PCN data access committee.

## Supporting information


Table S1:

## Data Availability

The data that support the findings of this study are available from the corresponding author upon reasonable request.
