# Supplementary material for: Risk of recurrent spontaneous preterm birth following preterm full dilatation cesarean delivery
Source: Acta Obstet Gynecol Scand. 2026 Jan 19;105(3):528–36. doi: 10.1111/aogs.70115 (PMC12942043; doi:10.1111/aogs.70115)
Supplement: Supplementary file 1 — Table S1: [file AOGS-105-528-s001.docx]

**Table S1: Preterm birth prophylactic intervention rates and recurrent spontaneous preterm birth according to mode of previous spontaneous preterm birth.**

|  | **Mode of previous spontaneous preterm birth** | | |  |
| --- | --- | --- | --- | --- |
|  | **FDCD** | **Vaginal Birth** | **CD<10cm** | **p-value** |
| **PBI** | 38.1 (8/21) | 25.9 (28/108) | 16.2 (18/111) | 0.046 |
| **Vaginal cerclage** | 19.0 (4/21) | 13.9 (15/108) | 9.0 (10/111) | 0.320 |
| **Vaginal progesterone only** | 19.0 (4/21) | 12.0 (13/108) | 7.2 (8/111) | 0.201 |
|  |  |  |  |  |
| **sLM or sPTB <37 weeks despite PBI** | 62.5 (5/8) | 32.1 (9/28) | 33.3 (6/18) | 0.413 |
| **sLM or sPTB <34 weeks despite PBI** | 50.0 (4/8) | 17.9 (5/28) | 22.2 (4/18) | 0.168 |
| **sLM or sPTB <37 weeks despite vaginal cerclage** | 75 (3/4) | 33.3 (5/15) | 50 (5/10) | 0.879 |
| **sLM or sPTB <34 weeks despite vaginal cerclage** | 75 (3/4) | 26.7 (4/15) | 30 (3/10) | 0.183 |
| **sLM or sPTB <37 weeks despite vaginal progesterone** | 50(2/4) | 30.8 (4/13) | 12.5 (1/8) | 0.323 |
| **sLM or sPTB <37 weeks with no PBI** | 23.1 (3/13) | 11.3 (9/80) | 11.8 (11/93) | 0.474 |

Data are given as % (n/N) or median (IQR-interquartile range).

PBI, preterm birth prophylactic intervention; CD<10cm, cesarean delivery at <10cm cervical dilatation; FDCD, full dilatation cesarean delivery; sLM, spontaneous late miscarriage (14-22 weeks gestation); sPTB, spontaneous preterm birth (≥23 weeks gestation).

**Table S2: Demographic characteristics of women with previous preterm full dilatation cesarean delivery who had transvaginal ultrasound measurements of their CD scar characteristics in the mid-trimester.**

| **Background characteristics** | **Previous preterm**  **FDCD**  **(n=15)** |
| --- | --- |
| Maternal age, years | 35.0 (32.0-37.0) |
| BMI, kg/m^2^ | 22.1 (20.9-25.9) |
| Ethnicity |  |
| -White | 46.7 (7) |
| -Black | 6.7 (1) |
| -South-east Asian | 13.3 (2) |
| -Other | 33.3 (5) |
| Current smoker | 0 (0) |
| Parity |  |
| 1 | 66.7 (10) |
| ≥2 | 33.3 (5) |
| Cervical surgery | 13.3 (2) |
| Uterine anomaly | 6.7 (1) |
| **Previous FDCD history** |  |
| Gestation at delivery, weeks | 32.6 (31.0-35.9) |
| Birthweight, g | 2030.5 (1563.8-2872.5; n=14) |
| Trial of instrumental delivery | 20.0 (3) |
| Duration of second stage of labor, hrs | 1.4 (0.8-4.0; n=6) |
| Uterine incision extension | 6.7 (1) |
| Cervical laceration | 6.7 (1) |
| Interpregnancy interval, months | 14.1 (11.0-37.2; n=11) |

Data are given as median (IQR- interquartile range) or % (n). BMI, body mass index; FDCD, full dilatation cesarean delivery.

**Table S3: Previous preterm full dilatation cesarean scar characteristics.**

| **CD scar characteristics** | **Previous Preterm FDCD**  **(n=15)** |
| --- | --- |
| Gestation of scar measurement, weeks | 17.1 (15.8-18.4) |
| Scar visualisation | 86.7 (13/15) |
| Presence of niche | 23.1 (3/13) |
| Scar distance to internal os, mm | 0.0 (0.0-6.4) |
| CD scar in cervix (at or below level of internal cervical os) | 53.8 (7/13) |
| CD scar in cervix or <5mm above internal os | 76.9 (10/13) |

Data are given as % (n/N) or median (IQR-interquartile range).

CD, cesarean delivery; FDCD, full dilatation cesarean delivery.
